# Predictors of Replacing Alcohol With Cannabis Among Adult Women

**DOI:** 10.7759/cureus.95821

**Published:** 2025-10-31

**Authors:** Jennifer Attonito, Jocelyn E Mueller, Karina Villalba

**Affiliations:** 1 Health Administration, Florida Atlantic University, Boca Raton, USA; 2 Psychology, University of California San Diego, San Diego, USA; 3 College of Medicine, University of Central Florida, Orlando, USA

**Keywords:** alcohol, cannabis, harm reduction, mental health, women

## Abstract

Background: Alcohol use among women varies by age, with younger women more likely to binge drink and older women more often engaging in consistent, long-term consumption. Both groups face health risks, including chronic disease, mental health conditions, and sleep disturbance. Cannabis has been proposed as a harm reduction substitute for alcohol because of its lower risks of dependency and health harms. The aims of this study are (a) to identify differences between younger and older women regarding their choices to use cannabis products as a substitute for alcohol and (b) to explore multiple drivers (sleep, stress, health state, post-traumatic stress disorder (PTSD), depression, and severity of alcohol use) behind the choice to replace alcohol with cannabis.

Methods: A cross-sectional online survey was conducted with 413 women aged 18 years and above who reported lifetime cannabis use. Participants were stratified into younger (<56 years) and older (≥56 years) groups. Measures included sociodemographics, cannabis substitution behaviors (cannabidiol (CBD), tetrahydrocannabinol (THC), or both), self-rated health, sleep and stress difficulties, and validated scales: Alcohol Use Disorders Identification Test (AUDIT), Primary Care PTSD Screen (PC-PTSD-4), Generalized Anxiety Disorder-7 (GAD-7), and Patient Health Questionnaire-8 (PHQ-8). Group differences were tested using chi-square and t-tests, and logistic regression identified predictors of substitution.

Results: Younger women (mean age 44.2 years) were significantly more likely than older women (mean age 62.9 years) to substitute THC for alcohol (14.0% vs. 7.8%, p = 0.019) and reported higher rates of sleep problems (52.5% vs. 39.1%, p = 0.007) and stress-coping difficulties (37% vs. 27%, p = 0.013). They also scored higher on AUDIT, PTSD, GAD, and PHQ instruments (all p < 0.01). Older women were more likely not to substitute cannabis for alcohol (83.5% vs. 71.0%, p = 0.002). Regression analyses showed that younger women with poorer health (OR = 1.76, 95% CI: 1.04-3.00) and higher AUDIT scores (OR = 1.07, 95% CI: 1.01-1.14) were more likely to substitute both CBD and THC. Sleep problems strongly predicted THC substitution in younger women (OR = 5.82, 95% CI: 1.58-21.45). Among older women, PTSD symptoms predicted substitution of both CBD and THC (OR = 1.60, 95% CI: 1.01-2.55), and sleep problems predicted THC substitution (OR = 3.05, 95% CI: 1.00-9.32).

Conclusions: Age-related differences emerged in women’s substitution of cannabis for alcohol. Younger women more frequently substituted THC and were influenced by alcohol severity, poor health, and sleep disturbance, whereas older women substituted less often, with PTSD and sleep difficulties as key predictors. These findings underscore cannabis substitution as a nuanced harm reduction strategy that requires age-specific approaches.

## Introduction

Excessive drinking among women differs significantly between younger and older age groups [1]. Younger women are more likely to engage in binge drinking, a behavior commonly influenced by factors such as socialization, peer pressure, and stress related to careers or relationships [2]. In contrast, older women who engage in excessive drinking may do so more consistently over time, often because of loneliness, chronic health issues, or coping with life transitions such as retirement or loss [3]. While younger women report higher instances of acute alcohol use, older women may face more severe long-term health consequences, including liver disease, cardiovascular problems, and cognitive decline, due to prolonged alcohol consumption [4]. For example, a review of national data from 2013 to the study’s publication in 2019 found that among young women aged 26-34, the prevalence of binge drinking rose from 20.8% to 25.9%, and among women aged 45-64, binge drinking increased from 9.5% to 13% [5].

Although drinking patterns differ by age, both younger and older women face significant health risks and may choose to reduce alcohol intake for health-related reasons such as chronic diseases, including liver and cardiovascular disease and cancers; mental health problems, including anxiety and depression; sleep difficulties; and the desire to improve quality of life. Prior research suggests that cannabis may serve as an acceptable replacement for alcohol as part of a harm reduction strategy [6]. While both alcohol and cannabis have psychoactive properties, cannabis compounds, particularly those that are cannabidiol (CBD) dominant, tend to have fewer harmful effects on health and behavior. The potential medical and psychiatric therapeutic effects of cannabis [7,8], combined with its lower risk of dependency [6] compared with alcohol, make it an appealing alternative. Moreover, cannabis does not carry the same level of risk for liver damage, cancer, impaired judgment, and dependency that alcohol does [9]. By substituting cannabis products for alcohol, women may reduce their risk of developing serious health conditions, decrease the likelihood of engaging in risky behaviors, and potentially experience improved overall well-being [6,10]. In a recent cross-sectional survey of individuals who substituted cannabis for alcohol, 30.9% stopped alcohol consumption entirely, while 36.7% reported reducing alcohol intake by at least 75%, with those experiencing negative psychological symptoms being more prone to substitution [11].

Chronic alcohol use is a common maladaptive coping mechanism for stress [12], which over time diminishes an individual’s resilience and exacerbates stress-related disorders. The relationship between anxiety disorders and alcohol use disorder is bidirectional; individuals with anxiety disorders may use alcohol to alleviate their symptoms, while alcohol use is also a known risk factor for developing anxiety disorders [13]. Similarly, post-traumatic stress disorder [14] and depression [15] have been proposed to follow the same self-medication pattern, as well as the reverse effect, in which alcohol use worsens symptoms [16]. Furthermore, sleep disturbance can serve as a pathway to increased alcohol use, in part because alcohol is often used as a sleep aid [17]. However, even acute alcohol consumption disrupts sleep throughout the night, and tolerance to its sedative effects develops quickly [18].

Previous research suggests that cannabis, particularly CBD, may help alleviate post-traumatic stress disorder (PTSD) symptoms and reduce fear responses and stress-related behaviors [19]. Another study found that cannabis users report significantly lower levels of depression and better overall quality of life [20]. Moreover, cannabinoids may help modulate sleep-wake cycles, improve sleep quality, and reduce sleep latency without the adverse effects of alcohol [17,21]. Tetrahydrocannabinol (THC)-dominant cannabis, in particular, may shorten the time needed to fall asleep and enhance slow-wave sleep, leading to more restorative sleep patterns [22]. Given the significant health risks associated with chronic alcohol use, the therapeutic potential of cannabis for addressing these issues presents a promising alternative for women.

This study evaluates differences between younger and older women regarding their choices to use cannabis products as a substitute for alcohol. In addition, investigators explore multiple factors behind the choice to replace alcohol with CBD, THC-containing marijuana, or both. Stratified by age, models were structured to test the following potential predictors for these substance choices: sleep problems, stress-coping difficulties, health state, PTSD, depression, and severity of alcohol use.

The aims of this study are (a) to evaluate differences between younger and older women regarding their choices to use cannabis products as a substitute for alcohol and (b) to explore multiple drivers behind the choice to replace alcohol with CBD, THC-containing marijuana, or both. Stratified by age, models were structured to test the following potential predictors for these substance choices: sleep problems, stress-coping difficulties, health state, PTSD, depression, and severity of alcohol use.

## Materials and methods

In this online cross-sectional study, we surveyed a cohort of 413 women ≥18 years old in Florida and Georgia who reported any alcohol and cannabis use in their lifetime (no power analysis conducted). Recruitment and data collection took place between April and September 2022. Qualtrics was used to recruit respondents from various sources, including website intercept recruitment, member referrals, targeted email lists, customer loyalty web portals, permission-based networks, and social media. All participants provided electronic informed consent before accessing the survey and were compensated via Qualtrics based on the time intensity of survey completion. In the survey, the term “marijuana” referred to cannabis products with THC, and “CBD” referred to cannabis products without THC. Participants were categorized based on age stratification at the median, with one group consisting of individuals aged ≥56 and another <56. The study protocol was approved by the University of Central Florida Institutional Review Board.

Measures

Nominal and categorical questions surveyed sociodemographic characteristics (age, Hispanic ethnicity, race, educational attainment, and employment status). In addition, de novo binary (yes/no) questions assessed whether respondents substituted CBD, THC, both, or neither for alcohol; whether they had difficulty sleeping (not being able to fall asleep/stay asleep, frequent napping); and whether they experienced trouble coping with stress more than usual. Another individual question asked respondents to rate their health in the past month on a scale of 1 = “excellent” to 6 = “very poor,” which was stratified by good-to-excellent (1-2) versus all else.

PTSD Screening Tool

The Primary Care PTSD Screen [23] is a 4-question screening tool used to identify individuals with probable PTSD in primary care settings and is available open access on the U.S. Department of State website. It assesses lifetime exposure to traumatic events and resulting symptomology. For diagnostic purposes, the results of the PC-PTSD-4 should be considered “positive” if a patient answers “yes” to any three of the four questions; the total number of “yes” responses was calculated for each case in this study to measure the degree of symptomology.

AUDIT Measure

Alcohol Use Disorders Identification Test (AUDIT) [24] was used to assess participants' alcohol consumption patterns and potential alcohol-related problems. Developed by the World Health Organization and available open access at https://nida.nih.gov/sites/default/files/files/AUDIT.pdf, AUDIT is a screening tool designed to detect harmful alcohol consumption. It consists of 10 questions covering various aspects of alcohol use, including frequency, quantity, and consequences of drinking. The instrument yielded a total score between 0 and 40 with good interitem reliability (α = 0.88).

GAD Measure

The Generalized Anxiety Disorder 7 (GAD) [25] scale is a self-report questionnaire designed to assess the severity of generalized anxiety disorder symptoms. This instrument is available open access as a figure in the primary source (https://www.phqscreeners.com). It consists of 7 items reflecting common symptoms experienced over the past two weeks. Each item is scored on a Likert scale from 0 to 3, with total scores ranging from zero to 21 and excellent reliability (α = 0.91).

PHQ-8 Measure

The Patient Health Questionnaire depression scale [26] is an eight-item self-report tool used to diagnose and measure the severity of depressive disorders. This instrument is available open access as an appendix in the primary source (citation provided). Each item asks respondents to report the frequency of experiencing common depressive symptoms on a scale of 0 = “Not at all” to 3 = “Nearly every day.” The instrument showed good interitem reliability (α = 0.86).

Data analysis

For categorical/binary measures, percentages were reported, and chi-square analyses were calculated to assess differences between groups. For continuous variables, means and standard deviations were reported, and independent samples t-tests were calculated to assess differences between groups. Probability values reported were two-tailed and set at p < 0.05. Six multivariate logistic regression models were tested in total; for each of the two groups of women, models predicted: (a) odds of substituting both forms of cannabis for alcohol, (b) odds of substituting CBD for alcohol, and (c) odds of substituting marijuana for alcohol. Predictors for all models included education, employment, sleep difficulties, health state, and stress coping difficulties, plus total PTSD, GAD, AUDIT, and PHQ scores. Model fit is reported as per Hosmer and Lemeshow tests. IBM SPSS Statistics for Windows, Version 27 (Released 2020; IBM Corp., Armonk, New York) was used to conduct statistical analyses.

## Results

Sociodemographics

The total sample for this study comprised 413 women aged 18 years or older who reported using cannabis in the past year. The younger (<56 years) group represented 44.3% of the sample, with a mean age of 44.2 (SD = 11.45). In this group, 9.3% identified as Hispanic, 77.6% reported their race as Caucasian, and 16.4% as Black/African American. Among older women (≥56 years), the mean age was 62.9 (SD = 5.65); 5.7% identified as Hispanic, 90% reported their race as Caucasian, and 7.4% as Black/African American. Regarding education and employment status, 74.9% of younger women versus 77.8% of older women reported having a college or trade school degree, while 56.3% of younger women versus 31.3% were employed (Table 1).

Bivariate observations

Significant differences between groups were observed on several measures. Older women were less likely than younger women to be employed (p < 0.001), more likely to report White as their race (p = 0.004), and more likely to report not having substituted cannabis for alcohol (83.5% vs. 71.0%; p = 0.002). Younger women were more likely than older women to substitute THC for alcohol (14.0% vs. 7.8%, p = 0.019), reported more difficulty sleeping (52.5% vs. 39.1%, p = 0.007), and reported greater difficulty coping with stress (37% vs. 27%, p = 0.013). They also yielded higher scores on the GAD (p < 0.001), PTSD (p < 0.001), PHQ (p < 0.001), and AUDIT (p = 0.006) instruments (Table 1).

**Table 1:** Univariate descriptive statistics and bivariate differences in sociodemographic factors and other health and mental health factors between older and younger women (N = 413) *Binary or categorical factors. ^†^Continuous factors. This table provides univariate statistics for all variables measured, stratified by age (<56 years v. 56+ years). Means/frequencies were compared between age groups and statistical significance is provided. CBD: cannabidiol, THC: tetrahydrocannabinol, GAD: generalized anxiety disorder, PTSD: post-traumatic stress disorder, PHQ: Patient Health Questionnaire, AUDIT: Alcohol Use Disorders Identification Test.

Variables	<56 years (N = 183; 44.3%)	≥56 years (N = 230; 55.7%)	p-value*
Age*	44.25 (11.45%)	62.9 (5.65%)	<0.001
Ethnicity Hispanic*	17 (9.3%)	13 (5.7%)	0.183
Race*			0.004
White	142 (77.6%)	207 (90%)	
Black/African American/Caribbean	30 (16.4%)	17 (7.4%)	
Other races	10 (5.5%)	6 (2.6%)	
At least college or trade school degree*	137 (74.9%)	179 (77.8%)	0.486
Employed (y/n)*	103 (56.3%)	72 (31.3%)	<0.001
Substituted cannabis for alcohol*			
Substituted CBD for alcohol	8 (4.4%)	4 (1.7%)	0.099
Substituted THC for alcohol	27 (14.8%)	18 (7.8%)	0.019
Substituted both CBD and THC for alcohol	18 (9.8%)	16 (7.0%)	0.19
Did not substitute either THC or CBD for alcohol	130 (71%)	192 (83.5%)	0.002
Difficulty sleeping (y/n)*	96 (52.5%)	90 (39.1%)	0.007
Difficulty coping with stress (y/n)*	69 (37.7%)	62 (27.0%)	0.013
Good-to-excellent health*	119 (65.0%)	143 (62.2%)	0.607
GAD^†^	10.12 (5.53%)	7.45 (5.12%)	<0.001
PTSD^†^	2.28 (1.54%)	1.7 (1.51%)	<0.001
PHQ^†^	10.68 (5.94%)	8.07 (4.98%)	<0.001
AUDIT^†^	5.46 (6.95%)	3.86 (4.85%)	0.006

Multivariate observations

All logistic regression models for both the younger and older groups displayed good fit according to the Hosmer and Lemeshow chi-square tests at p ≥ 0.05.

Substituting both CBD and THC for alcohol

As shown in Table 2, younger women were more likely to substitute both CBD and THC products for alcohol if they reported poorer health (OR = 1.76; 95% CI: 1.04, 3.00) and/or reported more severe alcohol problems (OR = 1.07; 95% CI: 1.01, 1.14). Sleep problems, stress coping, PTSD, depression, and anxiety were not significantly associated with substituting both cannabis products for alcohol among younger women. Older women were more likely to substitute CBD and THC for alcohol if they had higher PTSD scores (OR = 1.60; 95% CI: 1.01, 2.55) and/or reported more severe drinking problems (OR = 1.19; 95% CI: 1.09, 1.29). Sleep, stress coping, depression, anxiety, and health state were not significant predictors in the older group.

**Table 2:** Regression model testing sociodemographic and health/mental health factors predicting substitution of alcohol for both THC and CBD among younger (N = 183) and older (N = 230) women *Binary or categorical factors. ^†^Continuous factors. This table displays two regression models: substitution of alcohol for both CBD/THC among younger women and older women. Variables included in each regression model were: employment status, education status, sleep disturbance, stress coping, health state, as well as PTSD, AUDIT, PHQ, and GAD instruments. Model fit is provided for each regression analysis. Odds ratios and statistical significance are displayed for the variables in each model. CBD: cannabidiol, THC: tetrahydrocannabinol, PTSD: post-traumatic stress disorder, AUDIT: Alcohol Use Disorders Identification Test, PHQ: Patient Health Questionnaire, GAD: generalized anxiety disorder, SE: standard error, OR: odds ratio, CI: confidence interval.

	Younger				Older			
Variables	β	SE	OR (CI)	p	β	SE	OR (CI)	p
Substituting both CBD and THC	Model fit: chi-square = 4.20, p= 0.839				Model fit: chi-square = 3.99, p=0.857			
Employed*	0.077	0.597	1.08 (0.34, 3.48)	0.898	-0.261	0.676	0.77 (0.20, 2.90)	0.7
Education*	-0.076	0.247	0.93 (0.57, 1.51)	0.76	0.145	0.361	1.16 (0.57, 2.34)	0.688
Sleep problems*	0.972	0.645	2.64 (0.75, 9.36)	0.131	0.358	0.661	1.43 (0.39, 5.22)	0.588
Stress coping*	-1.126	0.686	0.32 (0.08, 1.24)	0.1	-1.164	0.872	0.31 (0.06, 1.72)	0.182
Health state*	0.564	0.269	1.76 (1.04, 3.00)	0.036	0.298	0.345	1.35 (0.68, 2.65)	0.389
PTSD^†^	0.191	0.23	1.21 (0.77, 1.90)	0.407	0.473	0.237	1.60 (1.01, 2.55)	0.049
AUDIT^†^	0.067	0.031	1.07 (1.01, 1.14)	0.029	0.171	0.044	1.19 (1.09, 1.29)	0
PHQ^†^	0.098	0.081	1.10 (0.94, 1.29)	0.229	-0.011	0.09	0.99 (0.83, 1.18)	0.9
GAD^†^	0.013	0.081	1.01 (0.86, 1.19)	0.869	0.003	0.081	1.00 (0.86, 1.18)	0.967

Substituting CBD for alcohol

Among younger women, reporting more severe alcohol problems was the only significant predictor for substituting CBD products for alcohol (OR = 1.10; 95% CI: 1.01, 1.19). Health state, sleep, stress coping, PTSD, depression, and anxiety were not significantly associated with CBD substitution. The older group did not yield any significant predictors for CBD substitution (Table 3).

**Table 3:** Regression model testing sociodemographic and health/mental health factors predicting substitution of alcohol for CBD among younger (N = 183) and older (N = 230) women *Binary or categorical factors. ^†^Continuous factors. This table displays two regression models: substitution of alcohol for CBD among younger women and older women. Variables included in each regression model were: employment status, education status, sleep disturbance, stress coping, health state, as well as PTSD, AUDIT, PHQ, and GAD instruments. Model fit is provided for each regression analysis. Odds ratios and statistical significance are displayed for the variables in each model. CBD: cannabidiol, PTSD: post-traumatic stress disorder, AUDIT: Alcohol Use Disorders Identification Test, PHQ: Patient Health Questionnaire, GAD: generalized anxiety disorder, SE: standard error, OR: odds ratio, CI: confidence interval.

	Younger				Older			
Variable	β	SE	OR (CI)	p	β	SE	OR (CI)	p
Substituting CBD	Model fit: chi-square = 7.91, p= 0.442				Model fit: chi-square = 9.23, p= 0.324			
Employed*	-1.535	1.201	0.21 (0.02, 2.26)	0.201	-0.723	1.077	0.48 (0.06, 4.01)	0.502
Education*	-0.388	0.358	0.68 (0.34, 1.37)	0.279	-0.253	0.587	0.78 (0.25, 2.45)	0.667
Sleep*	0.703	0.864	2.02 (0.37, 10.99)	0.416	-0.581	1.28	0.56 (0.04, 6.87)	0.65
Stress coping*	-1.88	1.251	0.15 (0.01, 1.77)	0.133	0.412	1.294	1.51 (0.12, 19.06)	0.75
Health state*	0.092	0.447	1.20 (0.46, 2.64)	0.836	0.512	0.643	1.67 (0.47, 5.88)	0.426
PTSD^†^	-0.023	0.324	0.98 (0.52, 1.84)	0.943	0.116	0.385	1.12 (0.53, 2.39)	0.763
AUDIT^†^	0.094	0.043	1.10 (1.01, 1.19)	0.028	0.059	0.098	1.06 (0.88, 1.28)	0.547
PHQ^†^	0.058	0.136	1.06 (0.81, 1.38)	0.67	-0.068	0.183	0.93 (0.65, 1.34)	0.708
GAD^†^	-0.073	0.129	0.93 (0.72, 1.20)	0.572	-0.07	0.174	0.93 (0.07, 1.31)	0.688

Substituting THC for alcohol

As shown in Table 4, among younger women, having sleep problems (OR = 5.82; 95% CI: 1.58, 21.45) and/or more severe alcohol problems (OR = 1.09; 95% CI: 1.03, 1.16) predicted a greater likelihood of substituting THC for alcohol. Health state, stress coping, PTSD, depression, and anxiety were not significantly associated with THC substitution. For older women, sleep was the only significant predictor for substituting THC for alcohol (OR = 3.05; 95% CI: 1.00, 9.32). No other factors were significantly associated with THC substitution in this group.

**Table 4:** Regression model testing sociodemographic and health/mental health factors predicting substitution of alcohol for THC among younger (N = 183) and older (N = 230) women *Binary or categorical factors. ^†^Continuous factors. This table displays two regression models: substitution of alcohol for THC among younger women and older women. Variables included in each regression model were: employment status, education status, sleep disturbance, stress coping, health state, as well as PTSD, AUDIT, PHQ, and GAD instruments. Model fit is provided for each regression analysis. Odds ratios and statistical significance are displayed for the variables in each model. THC: tetrahydrocannabinol, PTSD: post-traumatic stress disorder, AUDIT: Alcohol Use Disorders Identification Test, PHQ: Patient Health Questionnaire, GAD: generalized anxiety disorder, SE: standard error, OR: odds ratio, CI: confidence interval.

	Younger				Older			
Variables	β	SE	OR (CI)	p	β	SE	OR (CI)	p
Substituting THC	Model fit: chi-square = 9.36, p= 0.313				Model fit: chi-square = 11.52, p=0.079			
Employed*	0.55	0.509	1.17 (0.64, 4.70)	0.28	-0.259	0.545	0.77 (0.26, 2.24)	0.635
Education*	0.218	0.227	1.24 (0.80, 1.94)	0.337	0.153	0.281	1.16 (0.67, 2.02)	0.586
Sleep*	1.761	0.666	5.82 (1.58, 21.45)	0.008	1.115	0.57	3.05 (1.00, 9.32)	0.049
Stress coping*	0.449	0.535	1.57 (0.55, 4.47)	0.401	0.338	0.569	1.40 (0.46, 4.28)	0.552
Health state*	-0.159	0.234	1.85 (0.54, 1.35)	0.496	0.027	0.312	1.03 (0.56, 1.89)	0.93
PTSD^†^	0.002	0.167	1.00 (0.72, 1.39)	0.989	0.094	0.189	1.10 (0.76, 1.59)	0.621
AUDIT^†^	0.088	0.03	1.09 (1.03, 1.16)	0.003	-0.021	0.058	0.98 (0.87, 1.10)	0.722
PHQ^†^	0.072	0.074	1.07 (0.93, 1.24)	0.327	-0.007	0.082	0.99 (0.84, 1.17)	0.936
GAD^†^	-0.09	0.077	0.91 (0.78, 1.06)	0.244	0.016	0.081	1.01 (0.87, 1.19)	0.847

## Discussion

This study sought to identify multiple drivers behind women’s choices to replace alcohol with cannabis products, assuming that this choice reflected an effort toward harm reduction. Emerging research suggests that CBD may offer a viable alternative to alcohol for women seeking to reduce consumption without psychoactive effects [27]. As observed in previous research [28], this study found that younger women were substantially more likely than older women to substitute THC-containing cannabis for alcohol. Both groups were more likely to choose THC over CBD as a substitute for alcohol. In fact, only a small percentage of women opted for CBD as an alcohol substitute. It is possible that they sought the euphoric effects of a substance. Prior research has shown that marijuana can provide similar social and psychoactive functions as alcohol but with fewer negative consequences [6].

Severity of drinking emerged as the most frequent predictor for substituting CBD, THC, or both for alcohol, particularly among younger women, who also displayed significantly higher AUDIT scores. However, both age groups on average scored below the cutoff considered harmful or hazardous. Only 16.9% of older women and 24.1% of younger women scored eight or higher on this instrument. Studies have found that individuals who consume alcohol in larger quantities are more likely to view marijuana as a substitute [29]. The psychoactive effects of both substances may compel heavier drinkers to seek alternatives to manage intoxication or reduce alcohol-related harms.

Issues known to be associated with heavy alcohol use were examined as predictors. The strongest association observed was sleep, which predicted THC substitution. Younger women also reported more sleep problems than older women. Sleep problems can occur with heavier drinking and may act as a mediating factor between alcohol use and choosing to substitute another substance for alcohol [23]. Alternatively, women in this study may have attempted to use alcohol to treat sleep problems; however, cannabis products (depending on type and dosage) might be more effective in improving sleep quality [22].

Although younger women in this study were less likely than older women to report “good to excellent” health, poorer health was associated with substituting both THC and CBD products in the younger group. Some women may have been concerned about the negative health effects of drinking, making an alternative substance more compelling [6]. In other words, poorer health might mediate the relationship between alcohol use and the choice to use cannabis as a substitute. Further, younger women may not be as resilient against health problems as older women, making health challenges more distressing [30].

An unexpected finding in this study was that neither depressive nor anxiety symptoms predicted replacement of THC or CBD for alcohol in any multivariate model. Anxiety [13] and depression [15] are known to be linked with heavier drinking, a factor that predicted replacement in this study. It is recommended that future research test risky drinking as a mediating factor between anxiety or depression and the use of CBD or THC as an alcohol replacement.

Although older women in this study were less likely than younger women to report PTSD symptoms, it was nonetheless a predictor for substituting THC for alcohol in the older group. These findings can be interpreted separately or together. It is possible that younger and older women experience or interpret traumatic events differently, or that younger people with limited prior exposure to trauma may lack a reference point for handling traumatic events. Alternatively, irrespective of how trauma is experienced between groups, it is possible that the older group found THC-containing products to alleviate PTSD symptoms more effectively than alcohol [19].

Limitations

This study had distinct strengths. It included a relatively large sample of women from the southeastern United States and used several validated survey instruments; however, limitations must also be considered when interpreting the results. First, although validated instruments were used, self-report measures are subject to recall and social desirability bias, particularly when involving controversial or stigmatized topics. Second, the cross-sectional design precludes conclusions about causation. Additionally, although the sample was substantial, it may not be representative of all adult women who have used cannabis in the southeastern United States. Finally, it is important to note that the stratification of the sample at age 56 was an arbitrary division of “older” versus “younger” women, based entirely on the sample’s age distribution.

## Conclusions

The study by Chick and Nutt discussed above outlined several criteria for alcohol substitution therapies, including reducing harm, lowering the risk of dependency, and reducing the risk of misuse or overdose. Cannabis products may serve as a replacement based on these criteria; however, more research is necessary. A notable outcome of this study was that few respondents chose CBD as an alcohol substitute, suggesting that the desire for marijuana’s “high” remains prevalent among alcohol users. Emerging research suggests that CBD may offer a viable alternative to alcohol for women seeking to reduce consumption without psychoactive effects. CBD has been shown to possess anxiolytic and stress-relieving properties, which can be particularly beneficial for women who may use alcohol as a coping mechanism for stress and anxiety. Given the significant health risks associated with excessive alcohol consumption, CBD’s role as a safer, nonaddictive alternative warrants further investigation. For women, alcohol overuse presents heightened risks for liver disease, heart complications, and breast cancer. Thus, future studies on harm reduction approaches are warranted. This study explored cannabis substitution as a potential harm reduction strategy, and its findings may inform prevention and intervention efforts aimed at reducing alcohol-related harms and improving women’s health outcomes.
